# Dietary Inclusion of Blood Plasma with Yeast (*Saccharomyces cerevisiae*) Supplementation Enhanced the Growth Performance, Nutrient Digestibility, Lactobacillus Count, and Reduced Gas Emissions in Weaning Pigs

**DOI:** 10.3390/ani11030759

**Published:** 2021-03-10

**Authors:** Vetriselvi Sampath, Dong Heon Baek, Sureshkumar Shanmugam, In Ho Kim

**Affiliations:** 1 Department of Animal Resource and Science, Dankook University, No. 29 Anseodong, Cheonan, Choongnam 330-714, Korea; suve2314@gmail.com (V.S.); sureshbiogenetic@gmail.com (S.S.); 2 Department of Oral Microbiology and Immunology, Dankook University, No. 29 Anseodong, Cheonan, Choongnam 330-714, Korea; micro94@dankook.ac.kr

**Keywords:** blood plasma, *Sacchromyces cerevisiae*, weaning pigs, growth performance

## Abstract

**Simple Summary:**

Since 20th century, the use of yeast (*Saccharomyces cerevisiae*) supplement has been increased in animals feed due to its promising effects and potential to brighten the livestock industry in future. The feed cost of blood plasma is very expensive and, due to its high cost, it has been used mainly in piglet diets. In this regard, we implemented 3% blood plasma with 3% yeast supplementation to piglets mainly from 0–7 days, and gradually reduced the concentration of blood plasma with yeast supplement to 1.5:1.5% from 8–21 days and observed how it affects the overall performance of pigs on the remaining days 22–42 fed only basal diet. As expected, piglets fed blood plasma with yeast supplement over 0–21 days has a great impact on the growth performance, nutrient digestibility, fecal microbial, and gas emission at the end of the experiment. Thus, we suggest that blood plasma with yeast could be an excellent alternative in the livestock industry.

**Abstract:**

This experiment was performed to examine the hypothesis that blood plasma (BP) with yeast (*Saccharomyces cerevisiae)* supplement in the diet of weaning pigs could provoke the growth performance, nutrient digestibility, fecal microbial, and reduce harmful gas excretion. A total of one hundred and eighty healthy piglets were taken and assigned (complete random blocks) to three dietary treatments as: Phase 1: Treatment (TRT) 1-6% BP; TRT 2-3% BP + 3% yeast; TRT 3-6% yeast. Phase 2: TRT 1-3%; BP., TRT 2-1.5% BP + 1.5% yeast; TRT 3- 3% yeast. Phase 3: TRT 1- Control (CON) (Basal diet); TRT 2- CON; TRT 3- CON for six- weeks. Each treatment had twelve replicates and five (three gilts and two barrows) pigs per pen. Dietary inclusion of BP with yeast supplementation significantly increased the body weight of piglets during phase 2 (*p* = 0.003) and phase 3 (*p* = 0.032). In addition, TRT2 group piglets had a significant improvement in average daily gain at the end of each phase and overall (*p* = 0.047, 0.025, 0.018 and 0.012, respectively). At phase 3, TRT2 group piglets showed a significant improvement on nutrient digestibility of dry matter (*p* = 0.012) and nitrogen (*p* = 0.040). The fecal microbiota of TRT2 group piglets showed a tendency to increase the number of *Lactobacillus* counts at phase 1 (*p* = 0.07) and phase 2 (*p* = 0.06) as well as, a significant improvement at phase 3 (*p* = 0.021). In addition, TRT2 group piglets had trend to decrease NH_3_ (*p* = 0.074) and H_2_S (*p* = 0.069) during phase 2, and significantly reduced NH_3_ (*p* = 0.038) and H_2_S (*p* = 0.046) at phase 3. However, the fecal score of piglets remains unaffected during the entire trial. At the end of phase 1 piglets’ IgG (*p* = 0.008) was significantly increased with the inclusion of BP with yeast supplementation. Based on the positive effects on body weight, average daily gain, nutrient digestibility, *Lactobacillus* count, and reduced gas emission, we suggest that dietary supplement with BP and yeast in the diet of weaned piglet could serve as an excellent alternative to antibiotics growth promoters.

## 1. Introduction

Modern animal husbandry encounters several stressors that are associated with a negative impact on physiological, immunological, and biological properties of the animal’s gastrointestinal tract. Moreover, post-weaning diarrhea is one of the major problems for weaning piglets which leads to growth retardation, reduced food intake, and gastrointestinal disorders [1]. As well, post-weaning mortality could cause major economic losses to the livestock industries [2]. To tackle this situation, antibiotics as growth promoters (AGP) were routinely used by pig producers in order to promote growth performance and to prevent bacterial disease at the weaning stage [3]. However, over the past few decades, AGP use in livestock feedstuff has become the hottest debate among many researchers due to its bacterial resistance. Consequently, the European Union (EU) has restricted the use of antibiotics in the livestock industry since 2003 [4]. Several publications have pointed out a close link between antibiotic usage and bacterial resistance which cause health issues to consumers [5]. These anxieties have prompted scientists to discover a suitable alternative that could boost the quality of meat as well as livestock production. As a result, organic acids, probiotics, yeast, and phytogenic additives have been explored as an excellent source of substitutes for AGP in monogastric animal nutrition [4], since these additives have been adequately used in previous studies. However, the literature on the combined use of blood plasma and yeast supplements is still scarce, thus we used it as a potential alternative to suit swine diet.

Blood plasma (BP) is considered as one of the protein sources in animal feed [6], which is rich in vitamins and minerals such as vitamin B12, calcium, phosphorus, sodium, chloride, potassium, and magnesium. BP contains high levels of immunoglobulins (IgG) and plays a vital role in regulating the immune system and provides exclusive health benefits to young piglets. Furthermore, it is an excellent source of essential amino acids and superior to other protein sources such as skimmed milk [7]. Previously, Hansen et al. [8] and Kats et al. [9] pointed out that Spray-dried porcine plasma (SDPP) protein had improved the growth performance of piglets at week 1. Similarly, Jiang et al. [10] reported that the application of SDPP in post-weaning piglets’ diet had positively improved the growth performance, and feed intake. Additionally, Bosi et al. [11] stated that SDPP had enhanced the growth performance of piglet’s by reducing intestinal inflammation.

*Saccharomyces cerevisiae* (*S*. *cerevisiae)* yeast is rich in inositol (growth promoter), glutamic acid (contain palatability), and nucleotides [12]. The nucleotides in yeast are considered an essential nutrient for the rapid growth of animals. The use of *S*. *cerevisiae* yeast as a livestock feed additive has increased, especially after the ban on AGP [13]. Previously, Heugten et al. [14] and Trckova et al. [15] pointed out that a diet supplemented with live yeast (*S*. *cerevisiae*) had positively enhanced the performance of weaning piglets by stimulating their immune system. In addition, Desnoyers et al. [16] stated that S. *cerevisiae* has increased the feed efficiency and improve milk production as well milk quality in ruminants. The study of Shen et al. [17] showed that the administration of *S*. *cerevisiae* had significantly increased the villus heights and IgA levels in the ileum that aid in reducing postweaning diarrhea. The study of Pereira et al. [18] noted that the combined use of BP and yeast extract had reduced the negative impact on piglet performance during the post-weaning phase (0 to 21 days of age). Though BP in the animal diet provides high digestible and high-quality protein, due to its high cost, it has been used mainly in piglet diets. In this regard, the present study was conducted to determine whether a gradually reduced concentration of BP and yeast diet could enable the piglets to achieve similar growth performance, nutrient digestibility, fecal microbial, fecal gas emission, fecal score, and blood profile when fed basal diets.

## 2. Materials and Methods

### 2.1. Ethical Endorsement

The described protocols (Protocol number DK-1-1906) and weaning pigs used in this research were reviewed and approved by the Animal Care and Use Committee, Dankook University, Cheonan, South Korea.

### 2.2. Source of Yeast

Yeast (*S*. *cerevisiae)* supplement used in this study was commercially prepared in the name of NuPro^®^ and procured from Alltech Bio Korea co, Ltd. (Seoul, Republic of Korea). The active ingredients of yeast supplement were 3647 kcal/kg DE, 43.7% crude protein, 5.4% crude ash, 13% moisture, 70% pepsin digestibility.

### 2.3. Animal Husbandry and Dietary Regimens

This experiment was carried out in “the intensive pig farm” of “Dankook university” located at Jeonui (Sejong, South Korea) for 6 weeks with 180 crossbred weaning piglets ((Landrace Yorkshire) × Duroc)). Based on the initial average body weight (BW) (6.61 ± 0.93 kg) and sex, pigs were randomly divided into complete block pens and nutritional diets were offered during Phase 1 (0 to 7 days), Phase 2 (8 to 21 days) and, Phase 3 (22 to 42 days). The dietary treatments were

- Phase 1: TRT 1-6% BP; TRT 2-3% BP + 3% yeast; TRT 3-6% yeast.
- Phase 2: TRT 1-3% BP; TRT 2-1.5% BP + 1.5% yeast; TRT 3-3% yeast.
- Phase 3: TRT 1-CON (Basal diet); TRT 2- CON; TRT 3-CON.

Afore mentioned supplements were formulated according to the protocols of National Research Council (NRC) [19]. The dietary ingredients and the chemical composition of this trial are illustrated in Table 1. The experimental feed CON, BP, and yeast were mixed using DDK-801 feed mixer (Daedong Tech, Gyeonggi-do, Republic of Korea) and provided in mash form. Each treatment consisted of 12 replications with 5 pigs (three gilts and two barrows) per pen. The room temperature was maintained at 28 °C and every pen was equipped with self-feeder and nipple drinker which allow the piglets to enjoy free access to feed and water throughout the trial.

### 2.4. Sample Measurement and Laboratory Procedures

#### 2.4.1. Growth Performance

To calculate the performance variables, BW of individual piglets was determined using a GL-6000S balance machine (G-Tech International co., LTD., Gyeonggi-do, Republic of Korea) at beginning and end of each phase to determine the average daily gain (ADG). At the same time, the amount of feed consumed and leftovers (pen basis) were recoded to determine the average daily feed intake (ADFI), and feed conversion ratio (FCR).

#### 2.4.2. Nutrients Digestibility

Seven days prior to fecal collection, 0.2% chromic oxide (Cr2O3) was mixed in pigs’ diet. At the end of the experiment (day 42), the pig’s rectum was gently massaged by the trainer and fresh fecal samples were randomly collected from 24 piglets per treatment (2 pigs/pen), and immediately transported to the laboratory, and stored at −20 °C. Prior to analysis, freeze-dried samples were placed in a digital hot air-drying convection oven at 105 °C for 24 h. The samples were then taken out from the oven, pulverized well, and sieved using a 1 mm screen sieve. The nutrient digestibility of dry matter (DM), nitrogen (N), and gross energy (GE) analysis were carried out according to the guidelines of AOAC [20]. The chromium absorption was identified by UV-1201 spectrophotometry (Shimadzu, Kyoto, Japan) and the results were recorded for statistical analysis. 2 gm of feedand fecal sample was taken and placed in Parr 6400 (Parr Instrument Co., Moline, IL, USA) oxygen bomb calorimeter, the heat combustion of the samples was measured and recorded to determine GE. N was determined using a Tecator ^TM^ Kjeltec8400 (Hoganas, Sweden) analyzer. The total digestibility (cumulative result) was calculated using the equations of Upadhaya et al. [4]

#### 2.4.3. Microbial Shedding

At the end of each phase, fresh fecal samples were randomly collected from 24 piglets per treatment (2 pigs/pen) using micro-tubes and placed in sterile plastic bags. The samples were then placed in an insulated ice container and immediately taken to the research laboratory for microbial study. To confirm the existence of microbes, 1 gm of fresh fecal sample was taken and diluted with 9 mL of 1% peptone broth and mixed well with a vortex mixer. Then 0.02% of peptone solution was poured into MacConkey agar plates (Difco Laboratories, Detroit, MI, USA) and *Lactobacilli* medium III (DSMZ, Braunschweig, Germany) agar plates. The MacConkey agar plates were incubated at 37 °C for 1 day and *Lactobacilli* medium III agar plates were incubated at 39 °C, for 2 days. The bacterial colonies were counted immediately taken out from the incubator.

#### 2.4.4. Noxious Gas Emission

At the end of each phase, approximately 300 g of fecal samples were collected from 24 piglets per treatment, (2 pigs/pen) and placed in a plastic box (2600 mL) with a small hole in the middle and sealed with plaster. The samples were fermented for one day at room temperature (25 °C), and 100 mL of sample was taken from the headspace (approximately 2.0 cm) above the fecal sample for air circulation. Later the box was re-sealed to measure the fecal noxious content. Collected fecal samples were manually shaken for around 30 s to measure the crust formation on the surface and homogenized. Concentrations of NH_3_, H_2_S, CO_2_, Acetaldehyde, propionic acid, and acetic acid were measured within the scopes of 5.0 to 100.0 ppm (No. 3La, detector tube; Gastec Corp, Kanagawa, Japan) and 2.0 to 20.0 ppm (4LK, detector tube; Gastec Corp, Kanagawa, Japan).

#### 2.4.5. Fecal Score

At initial and end of each phase the fecal score was evaluated and recorded to determine the average value of 5 pigs/pen based on Hu et al.’s [21] scoring system: 1 = hard, dry pellets in a small, hard mass; 2 = hard, formed stool that remains firm and soft; 3 = soft, formed and moist stool that retains its shape; 4 = soft, unformed stool that assumes the shape of the container; 5 = watery, liquid stool that can be poured.

#### 2.4.6. Blood Profile

At initial and end of each phase, 5 mL of the blood sample was collected by puncturing jugular vein of 24 piglets per treatment (2 pigs/pen) using a sterilized syringe and stored in (K_3_EDTA) (Becton, Dickinson and Co., Franklin Lakes, NJ, USA) heparinized tubes without anticoagulant for blood urea nitrogen (BUN) analysis and non-heparinized tubes for serum creatinine analysis. The samples were centrifuged at 3000 rpm for 15 min at 4 °C. BUN was analyzed using the Abbott Spectrum urea nitrogen testing kit (Series II, Abbot Laboratories, Dallas, TX, USA). Creatinine concentrations were determined using an Astra-8 Analyzer (Beckman Instruments, Inc., Brea, CA 92621, USA). The IgG, IgM and IgA in serum were determine using an ELISA kit (Quantitation Kit; Bethyl Laboratories, Montgomery, TX, USA). The White blood cell, Red blood cell and lymphocyte counts were determined using Neubauer counting chamber [22].

### 2.5. Statistical Process

All data were subjected to the statistical analysis in a randomized complete block design using the general linear model procedures of SAS (SAS Inst. Inc., Carry, NC, USA), and the pen was used as the experimental unit. The initial body weight was used as a covariate for ADG. Before carrying out statistical analysis of the microbial counts, logarithmic conversion of the data was performed. The significance of differences between means was determined using Tukey’s test. *p* < 0.05 was considered as significant, *p* < 0.10 was considered as a trend.

## 3. Results

Dietary inclusion of BP with yeast supplementation had significantly improved the BW of piglets (*p* = 0.043; 0.032) during phases 2 and 3, respectively. Though TRT2 group piglets had a significant improvement in ADG at the end of each phase and overall (*p* = 0.047, 0.025, 0.018, and 0.012, respectively), the ADFI and FCR remain unaffected throughout the trial (Table 2). At the end of the experiment, TRT2 group piglets showed a significant improvement in nutrient digestibility of DM (*p* = 0.012) and N (*p* = 0.040) and no effects on E (Table 3). The fecal microbiota of TRT2 group piglets showed a tendency to increase the number of *lactobacillus* counts at phases 1 and 2 (*p* = 0.079, 0.061) as well as a significant improvement at phase 3 (*p* = 0.021); the *E. coli* remains unaffected (Table 4). Though TRT2 group piglets tended to decrease NH_3_ (*p* = 0.074) and H_2_S (*p* = 0.069) at phase 2, and significantly reduced NH_3_ (*p* = 0.038) and H_2_S (*p* = 0.046) at phase 3, they did not show any significant difference on acetaldehyde, CO_2_, propionate, acetic acid, and propionic acid throughout the trial (Table 5). Furthermore, there were no significant effects observed on the fecal score of weaning pigs fed BP with yeast supplementation during the entire trial (Table 6). A significant result was observed on piglets’ IgG (*p* = 0.008) with the inclusion of BP with yeast supplementation during phase 1. Moreover, there were no significant effects observed on WBC, RBC, Lymphocyte, IgM, IgA, BUN, and creatinine during the overall experiment (Table 7).

## 4. Discussion

Earlier research proved BP as a high- quality protein source in the diet of weaned piglets. The study of Pan et al. [23] pointed out that SDPP in the diet of weaning pigs had reduced intestinal disorders. In addition, Carlson et al. [24] stated that the inclusion of yeast supplement had increased the feed intake and daily gain of weaning piglets. In addition, Lawrence et al. [25] reported that plasma protein has a high impact on biological and immunoglobulin on nursing piglets. Apart from this, many researchers noticed that the presence of glutamic acid and peptides in yeast extracts (palatable properties), had improved the feed intake of pigs [26,27,28]. However, in the present study, we used BP with yeast in the diet of weaning pigs to enhance their growth performance. Expectedly, the results agreed with our hypothesis that BP with yeast supplementation had significantly improved BW and ADG of weaning piglets. This result was consistent with Pereira et al. [18] who observed that graded levels of yeast extract with blood plasma supplementation had significantly increased BW of weaned pigs. In 2015, Li et al. [29] noted that the growth performance of BW and ADG were significantly enhanced in piglets fed yeast extract supplementation. Similarly, Waititu et al. [30] stated that yeast extract with enzyme supplementation had improved ADG. The above-mentioned finding was consistent with our result that BP with yeast supplement had significantly increased ADG. However, a dietary supplement with BP and yeast had failed to show a positive effect on ADFI and this finding was correlated with a study by Costa [12] who observed that the inclusion of 6% yeast extract in the diet of piglets produced no significant variation in feed intake. Similarly, Rigueira [31], also found no significant changes in the feed intake of piglets (7 to 28 days of age) fed plasma with yeast extract (2.0% BP; 2.0% 2) supplement. As we all know, yeast extract could enhance the appetite [24] and the glutamic acid presence in yeast plays a vital role in increasing the palatability of diet and protecting the intestinal mucosa [32]. In this sense, we assume that no effects on ADFI might be due to the presence of low glutamic level in the experimental diet.

In 2012, Pereria et al. [18] demonstrate that yeast-derived protein had enriched with free amino acids, short-chain peptides, nucleotides, and inositol that could be proposed as a superior protein ingredient and act as an alternative to SDPP to suit weaning pigs’ diet. Previously, Yu [32] stated that yeast culture supplementation has not influenced the nutrient digestibility of DM in livestock animals. However, in the current study piglets fed BP with yeast supplementation had significantly improved the nutrient digestibility of DM and N and this finding agreed with the study of Shi and Kim [33] who reported that the ATTD of DM and N were significantly improved in weaner pigs fed with yeast extract culture supplementation. In 2017, Waititu et al. [34] stated that yeast extract or yeast products with blood plasma supplementation had positively affected intestinal morphology and functions, thereby increasing the digestibility of nutrients. To further support this data the study of Li and Kim [35] demonstrate that the inclusion of yeast extract supplementation had enhanced nutrient digestibility. We assume that the increased digestibility might be due to the presence of IgG in BP which helps to improve the immune system as well the presence of acetogens and antimicrobial compounds in yeast that prevent the pathogens and enhance the nutrient utilization.

The intestinal microbes play a major role in detoxifying harmful substances, preventing colonization of pathogens, recycling the nitrogen, and synthesis microorganisms of vitamins [36]. An increased *Lactobacillus* population in piglets could positively affect the host gut, whereas increased *E. coli* counts lead to negative impact [37]. Previously, Price et al. [38] suggested that *S. cerevisiae* fermentation product has altered the composition of the gastrointestinal microbial community, thereby increasing the population of *Lactobacillus* weaning pigs. The current study reveals that the *Lactobacillus* population was significantly increased, whereas *E. coli* counts remain unaffected with the inclusion of blood plasma and yeast supplementation; this finding was constant with Liu et al. [39] who stated that the pigs fed with nucleotides derived from *S. cerevisiae* had improved fecal *Lactobacillus* counts and reduced *E. coli* counts, which can contribute to enhanced metabolism of nutrients and intestinal morphology in pigs [34]. However, Sweeney et al. [40] stated that weaning pigs fed *S. cerevisiae* related β-glucans supplementation had significantly reduced *E. coli* counts without affecting *Lactobacillus* counts. Ammonium and hydrogen sulfide (harmful) are considered as the major hazardous gases of pig manure which lead to environmental pollution [41]. The elevated level of NH_3_ and H_2_S not only cause a health risk to animals but also to the workers [42]. Such hazardous gases as NH_3_ and H_2_S were significantly reduced in piglets fed blood plasma with yeast supplementation and this result correlated with Li and Kim [35], who observed a decreased H_2_S emissions in growing pigs fed dietary supplementation with *S. cerevisiae* cell-wall product. Fecal gas emission content is generally associated with nutrient digestibility and increased digestion which may lower substrates of microbial fermentation in the large intestine and resulted in reduced gas emission content [43]. In this sense, we assume that increased nutrient absorption in the intestine of piglets might be the reason for the reduced gas emission.

Few studies have been demonstrated that the combination of blood plasma with yeast diet has a beneficial effect on immune function. In 2016, studies of Waititu et al. [30] showed that serum urea nitrogen in weaning pig has been increased by hydrolyzed yeast diet. Furthermore, Zhang et al. [44] reported that the yeast extract had decreased the blood cholesterol content in pigs. While searching for the literature on the influence of yeast in animals blood profiles, we found that dietary supplementation with yeast could enhance the growth hormones (GH) and the immune response [42]. In 2009, Kim et al. [45] stated that the dietary inclusion of 1.0% of yeast in rats’ diet has enhanced its GH level. Creatinine levels in the blood may increase during periods of diarrhea as a result of the accumulation of protein deposits from the muscle to compensate for poor nutrient intake and/or absorption [46]. However, in this study the serum creatinine levels remain unaffected and thereby no effects were observed on the fecal score. Moreover, in this study, except for IgG, there were no significant effects observed (among treatments) on blood profiles of piglets fed BP with yeast supplement. To date, the influence of supplementing BP with yeast in weaning piglets, obtain a high effect on blood profile has not been reported. Thus, comparisons could not be made with other studies.

The feed cost of blood plasma is very expensive and, due to this high cost, in this study piglets were allowed to consume blood plasma with yeast supplement from 0–21 days, whereas from 22–42 days they were fed only basal diet. As expected, piglets being fed blood plasma with yeast supplement over 0–21 days had a great impact on the growth performance, nutrient digestibility, fecal microbial, and gas emission at the end of the experiment. However, the fecal score and blood profile were not adversely affected. Based on the positive impact we concluded that the combined use of BP with yeast supplementation could be an excellent alternative to antibiotics growth promoters in the livestock industry.

## Figures and Tables

**Table 1:** Composition of weaning pig diets (as fed-basis).

Item	Phase 1 (0–7 d)			Phase 2 (8–21)			Phase 3 (22–42 d)
	TRT1	TRT2	TRT3	TRT1	TRT2	TRT3	
Ingredients, %							
Corn	39.22	36.31	33.29	52.48	50.98	49.51	59.29
Soybean meal	16.42	18.92	21.45	16.60	17.87	19.12	22.46
Fermented soybean meal	5.00	5.00	5.00	4.00	4.00	4.00	3.00
SDPP	6.00	3.00	-	3.00	1.50	-	-
Yeast	-	3.00	6.00	-	1.50	3.00	-
Tallow	2.49	2.92	3.39	2.53	2.76	2.98	2.49
Lactose	13.46	13.46	13.46	7.78	7.78	7.78	3.18
Sugar	3.00	3.00	3.00	3.00	3.00	3.00	3.00
Whey protein	11.00	11.00	11.00	7.00	7.00	7.00	3.00
Monocalcium phosphate	0.90	0.86	0.82	1.08	1.06	1.04	1.14
Limestone	1.17	1.16	1.18	1.20	1.20	1.20	1.22
Salt	0.20	0.20	0.20	0.10	0.10	0.10	0.10
Methionine (99%)	0.22	0.21	0.21	0.15	0.15	0.15	0.08
Lysine	0.49	0.53	0.57	0.65	0.67	0.69	0.61
Mineral mix ^1^	0.20	0.20	0.20	0.20	0.20	0.20	0.20
Vitamin mix ^2^	0.20	0.20	0.20	0.20	0.20	0.20	0.20
Choline (25%)	0.03	0.03	0.03	0.03	0.03	0.03	0.03
Total	100.00	100.00	100.00	100.00	100.00	100.00	100.00
**Calculated value**							
Crude protein, %	20.00	20.00	20.00	18.00	18.00	18.00	18.00
Ca, %	0.80	0.80	0.80	0.80	0.80	0.80	0.80
P, %	0.60	0.60	0.60	0.60	0.60	0.60	0.60
Lys, %	1.60	1.60	1.60	1.50	1.50	1.50	1.40
Met, %	0.48	0.48	0.48	0.40	0.40	0.40	0.35
Metabolic Energy, kcal/kg	3450	3450	3450	3400	3400	3400	3350
FAT, %	4.18	4.55	4.94	4.65	4.84	5.03	4.89
Lactose, %	20.00	20.00	20.00	12.00	12.00	12.00	5.00

^1^ Provided per kg diet: Fe, 100 mg as ferrous sulfate; Cu, 17 mg as copper sulfate; Mn, 17 mg as manganese oxide; Zn, 100 mg as zinc oxide; I, 0.5 mg as potassium iodide; and Se, 0.3 mg as sodium selenite. ^2^ Provided per kilograms of diet: vitamin A, 10,800 IU; vitamin D3, 4000 IU; vitamin E, 40 IU; vitamin K3, 4 mg; vitamin B1, 6 mg; vitamin B2, 12 mg; vitamin B6, 6 mg; vitamin B12, 0.05 mg; biotin, 0.2 mg; folic acid, 2 mg; niacin, 50 mg; D-calcium pantothenate, 25 mg.

**Table 2:** Effect of blood plasma and yeast supplementation on the growth performance of weaning pigs ^1^.

Items	TRT1 ^1^	TRT2 ^1^	TRT3 ^1^	SEM ^2^	*p*-Value
Body weight, kg					
Initial	6.61	6.61	6.61	0.002	1.000
Phase 1	8.24	8.53	7.95	0.42	0.107.
Phase 2	13.98	14.27	12.35	0.23	0.043
Phase 3	24.89	27.14	23.70	0.38	0.032
Phase 1 (0–7 days)					
ADG, g	197	206	193	0.433	0.047
ADFI, g	214	219	199	10	0.913
FCR	1.112	1.120	1.109	0.015	0.321
Phase 2 (8–21 days)					
ADG, g	321	358	314	0.14	0.025
ADFI, g	433	415	436	15	0.741
FCR	1.380	1.363	1.399	0.022	0.428
Phase 3 (22–42 days)					
ADG, g	566	579	541	0.16	0.018
ADFI, g	791	790	785	17	0.583
FCR	1.467	1.479	1.491	0.015	0.169
Overall					
ADG, g	437	444	407	0.9	0.012
ADFI, g	579	577	564	10	0.844
FCR	1.424	1.417	1.437	0.013	0.347

^1^ Abbreviation: Phase 1: TRT1, 6% blood plasma (BP); TRT2, 3% BP+ 3% yeast; TRT3, 6% yeast. Phase 2: TRT1, 3% BP; TRT2, 1.5% BP + 1.5% yeast; TRT3, 3% yeas.t Phase 3: TRT1; TRT2; TRT3, Basal diet. (CON). ^2^ Standard error of means.

**Table 3:** Effect of blood plasma and yeast supplementation on nutrient digestibility of weaning pigs ^1^.

Items, %	TRT1 ^1^	TRT2 ^1^	TRT3 ^1^	SEM ^2^	*p*-Value
Dry matter	83.97	85.45	82.95	0.53	0.012
Nitrogen	81.59 ^b^	82.93 ^a^	80.67 ^ab^	0.66	0.040
Energy	80.72	82.73	81.01	0.64	0.077

^1^ Abbreviation: Phase 1: TRT1, 6% BP; TRT2, 3% BP+ 3% yeast; TRT3, 6% yeast. Phase 2: TRT1, 3% BP; TRT2, 1.5% BP + 1.5% yeast; TRT3, 3% yeast. Phase 3: TRT1; TRT2; TRT3, Basal diet. (CON). ^2^ Standard error of means. ^a,b^ Means in the same row with different superscript differ significantly (*p* < 0.05).

**Table 4:** Effect of blood plasma and yeast supplementation on the fecal microbial profile of weaning pigs ^1^.

Items, log_10_cfu/g	TRT1 ^1^	TRT2 ^1^	TRT ^1^	SEM ^2^	*p*-Value
Phase 1 (0–7 days)					
*Lactobacillus*	7.35	7.40	7.37	0.04	0.079
*E. coli*	6.02	5.42	5.03	0.09	0.210
Phase 2 (8–21 days)					
*Lactobacillus*	7.50	7.58	7.44	0.29	0.061
*E. coli*	6.76	5.68	5.14	0.31	0.275
Phase 3 (22–42 days)					
*Lactobacillus*	7.61	7.69	7.52	0.05	0.021
*E. coli*	7.14	6.19	5.30	0.07	0.485

^1^ Abbreviation: Phase 1: TRT1, 6% BP; TRT2, 3% BP+ 3% yeast; TRT3, 6% yeast. Phase 2: TRT1, 3% BP; TRT2, 1.5% BP + 1.5% yeast; TRT3, 3% yeast. Phase 3: TRT1; TRT2; TRT3, Basal diet (CON). ^2^ Standard error of means.

**Table 5:** Effect of blood plasma and yeast supplementation on the gas emission of weaning pigs ^1^.

Items, ppm	TRT1 ^1^	TRT2 ^1^	TRT3 ^1^	SEM ^2^	*p*-Value
Phase 1 (0–7 days)					
NH_3_	10.0	9.3	8.9	0.2	0.572
H_2_S	3.5	2.7	2.8.0	0.5	0.145
Acetaldehyde	2.5	2.8	2.4	0.3	0.617
CO_2_	1375	1325	1400	248	0.837
Acetic acid	2.3	2.0	1.7	0.4	0.334
Propionic acid	1.7	1.5	1.2	0.4	0.101
Phase 2 (8–21 days)					
NH_3_	10.8	9.8	11.4	0.1	0.074
H_2_S	1.7	1.5	2.3	0.5	0.069
Acetaldehyde	2.4	2.3	2.9	0.5	0.694
CO_2_	1200	1375	1275	272	0.995
Acetic acid	1.5	2.0	1.9	0.6	0.743
Propionic acid	2.4	1.5	2.2	0.4	0.342
Phase 3 (22–42 days)					
NH_3_	11.9	10.6	12.4	0.2	0.038
H_2_S	4.1	3.2	4.3	1.0	0.046
Acetaldehyde	2.5	2.4	2.6	0.6	0.732
CO_2_	1275	1375	1475	207	0.342
Acetic acid	2.3	2.1	1.5	0.5	0.669
Propionic acid	2.3	2.6	1.5	0.6	0.801

^1^ Abbreviation: Phase 1: TRT1, 6% BP; TRT2, 3% BP+ 3% yeast; TRT3, 6% yeast. Phase 2: TRT1, 3% BP; TRT2, 1.5% BP + 1.5% yeast; TRT3, 3% yeast. Phase 3: TRT1; TRT2; TRT3, Basal diet (CON). ^2^ Standard error of means.

**Table 6:** Effect of blood plasma and yeast supplementation on the fecal score of weaning pigs ^1^.

Items	TRT1 ^1^	TRT2 ^1^	TRT3 ^1^	SEM ^2^	*p*-Value
Fecal score ^3^					
Initial	3.55	3.53	3.51	0.03	0.548
Phase 1 (0–7 days)	3.51	3.46	3.48	0.03	0.281
Phase 2 (8–21 days)	3.36	3.32	3.34	0.04	0.133
Phase 3 (22–42 days)	3.26	3.24	3.21	0.05	0.816

^1^ Abbreviation: Phase 1: TRT1, 6% BP; TRT2, 3% BP+ 3% yeast; TRT3, 6% yeast. Phase 2: TRT1, 3% BP; TRT2, 1.5% BP + 1.5% yeast; TRT3, 3% yeast. Phase 3: TRT1; TRT2; TRT3, Basal diet (CON). ^2^ Standard error of means. ^3^ Score: 1 = hard, dry pellets in a small, hard mass; 2 = hard, formed stool that remains firm and soft; 3 = soft, formed, and moist stool that retains its shape; 4 = soft, unformed stool that assumes the shape of the container; 5 = watery, liquid stool that can be poured.

**Table 7:** Effect of blood plasma and yeast supplementation on the blood profile of weaning pigs ^1^.

Items	TRT1 ^1^	TRT2 ^1^	TRT3 ^1^	SEM ^2^	*p*-Value
Initial					
WBC, 10^3^/μL	13.90	14.02	15.82	1.99	0.903
RBC, 10^6^/μL	6.31	6.22	6.27	0.31	0.750
Lymphocyte, %	42.0	42.5	41.6	2.5	0.463
IgG, mg/dL	137.0	136.3	142.8	24.5	0.501
IgM, mg/dL	59.0	60.5	62.3	14.2	0.661
IgA, mg/dL	36.3	32.5	39.8	3.9	0.440
BUN, mg/dL	4.3	4.5	4.5	1.2	0.336
Creatinine, mg/dL	0.67	0.69	0.70	0.05	0.132
Phase 1 (0–7 days)					
WBC, 10^3^/μL	14.20	13.28	13.49	1.31	0.971
RBC, 10^6^/μL	6.63	6.68	6.40	0.23	0.526
Lymphocyte, %	44.0	43.6	42.7	4.1	0.942
IgG, mg/dL	170.3	172.8	182.3	7.2	0.008
IgM, mg/dL	82.5	66.8	83.8	10.5	0.292
IgA, mg/dL	65.8	73.5	59.3	4.2	0.247
BUN, mg/dL	6.3	6.8	6.5	1.1	0.197
Creatinine, mg/dL	0.78	0.79	0.85	0.06	0.634
Phase 2 (8–21 days)					
WBC, 10^3^/μL	15.36	17.28	18.64	1.32	0.128
RBC, 10^6^/μL	6.91	6.64	6.20	0.27	0.305
Lymphocyte, %	44.3	43.1	45.3	5.2	0.961
IgG, mg/dL	272.5	293.8	297.5	36.7	0.747
IgM, mg/dL	81.8	79.3	74.3	11.3	0.957
IgA, mg/dL	145.0	127.8	137.3	9.4	0.758
BUN, mg/dL	10.0	9.3	9.5	1.8	0.842
Creatinine, mg/dL	1.09	0.93	1.05	0.08	0.642
Phase 3 (22–42 days)					
WBC, 10^3^/μL	16.57	17.22	19.04	1.94	0.799
RBC, 10^6^/μL	6.61	6.46	6.72	0.35	0.902
Lymphocyte, %	44.0	46.0	47.2	3.3	0.536
IgG, mg/dL	285.0	304.3	309.8	25.4	0.437
IgM, mg/dL	84.0	86.3	81.8	10.1	0.760
IgA, mg/dL	328.0	310.0	316.8	9.7	0.258
BUN, mg/dL	16.8	15.5	14.5	2.9	0.992
Creatinine, mg/dL	1.07	1.06	0.94	0.07	0.430

^1^ Abbreviation: Phase 1: TRT1, 6% BP; TRT2, 3% BP+ 3% yeast; TRT3, 6% yeast. Phase 2: TRT1, 3% BP; TRT2, 1.5% BP + 1.5% yeast; TRT3, 3% yeast. Phase 3: TRT1; TRT2; TRT3, Basal diet (CON). ^2^ Standard error of means.

## Data Availability

Based on reasonable request, data sets of this study will be available from the corresponding author.
